# Ciliate Grazing on the Bloom-Forming Microalga *Gonyostomum semen*

**DOI:** 10.1007/s00248-024-02344-9

**Published:** 2024-01-18

**Authors:** Ingrid Bergman, Eva S. Lindström, Ingrid Sassenhagen

**Affiliations:** 1 Department of Ecology and Genetics/Limnology, Uppsala University, Uppsala, Sweden; 2 Biological Oceanography, Institute for Baltic Sea Research in Warnemünde, Seestraße 15, Rostock, 18119 Germany

**Keywords:** Predator–prey interaction, *Urotricha pseudofurcata*, Grazing experiment, Humic lakes

## Abstract

The freshwater raphidophyte *Gonyostomum semen* forms extensive summer blooms in northern European humic lakes. The development of these blooms might be facilitated by a lack of natural top-down control, as few zooplankton species are able to prey on these large algal cells (up to 100 μm) that expel trichocysts upon physical stress. In this study, we describe a small ciliate species (< 17 μm) that preys on *G. semen* by damaging the cell membrane until cytoplasm and organelles spill out. Sequencing of clonal cultures of the ciliate tentatively identified it as the prostomatid species *Urotricha pseudofurcata*. Grazing experiments illustrated that feeding by *U.* cf. *pseudofurcata* can significantly reduce cell concentrations of the microalga. However, differences in cell size and growth rate between two investigated ciliate strains resulted in noticeably different grazing pressure. Environmental sequencing data from five different lakes supported potential interactions between the two species. *Urotricha* cf. *pseudofurcata* might, thus, play an important role in aquatic ecosystems that are regularly dominated by *G. semen*, reducing the abundance of this bloom-forming microalga and enabling transfer of organic carbon to higher trophic levels.

**Supplementary Information:**

The online version contains supplementary material available at 10.1007/s00248-024-02344-9.

## Introduction

*Gonyostomum semen* is a globally distributed, freshwater phytoplankton species [1, 2]. Due to climatic and anthropogenic changes resulting in browning and warming of lake waters in Northern Europe, the species has increased in abundance [3–5]. *G. semen* regularly forms extensive summer blooms in humic lakes with a pH < 7, representing more than 95% of all phytoplankton cells and dominating the community [6, 7]. *G. semen* possesses trichocysts, which discharge mucilaginous strands upon physical stimulation. These strands or potential associated chemicals cause skin irritation for people bathing in the lake and clog filters in water treatment plants [6, 8].

The dominance of *G. semen* might be facilitated by a lack of natural top–down control, as previous studies have shown that only large cladocerans and copepods ingest *G. semen* [9, 10]. This lack of grazers is surprising, as *G. semen* can be considered a good food source due to its comparatively high fatty acid content [11, 12]. Furthermore, this microalgal species does not possess a sturdy cell wall, but only a cell membrane, which breaks easily upon physical stress [8]. Its inedibility might be related to its trichocyst ejections reaching up to 200 μm [6, 8], as well as its large cell size (up to 100 μm in length), making *G. semen* too big for ingestion by most zooplankton species [13, 14]. Therefore, in cases of absence of large zooplankton [9], the organic carbon generated by primary production from *G. semen* might not directly be transported to higher trophic levels of the food web, but rather get remineralized by the microbial loop or buried in the sediment upon cell death.

Ciliates constitute an important component of the microzooplankton community, but are generally assumed to have a preference for small-sized prey (< 20 μm) [15, 16]. Nevertheless, they can consume a large amount of phytoplankton biomass and represent key players in aquatic food webs due to their ability to exploit various food sources [17]. The prostomatid genus *Urotricha* represents one of the most common freshwater plankton ciliates [18], which can reach cell concentrations of a few 100 cells/mL. Prostomatid ciliates are generally known to graze bacteria and picocyanobacteria [16, 19], while *Urotricha* sp. has been reported to prey intensely on small cryptophytes [15, 20]. Furthermore, the prostomatid *Coleps* can act as a scavenger and feed on dead organic matter [21]. Ciliates contribute therefore to the carbon flux from small primary producers to higher trophic levels in aquatic ecosystems [14, 22]. However, besides low numbers of predator–prey studies, information about the autecology of most prostomatid ciliates is still very limited [18].

In this study, we report on a small prostomatid ciliate feeding on the large freshwater raphidophyte *G. semen*. Using laboratory-based grazing experiments, we aimed to assess the impact of this ciliate on cell concentration and population growth of the phytoplankton species. Sequencing data from environmental plankton communities were investigated to determine the co-occurrence of the ciliate species and *G. semen* in different lakes. We hypothesized that the ciliate will exert significant top-down control on *G. semen*.

## Materials and Methods

### Establishment of Cultures

Roughly every week from June to September 2020, plankton net samples with a mesh size of 20 μm were collected from the open water from a boat in Stora Hålsjön (59.989502N, 17.091729E), from piers in Siggeforasjön (59.977783N, 17.146841E) and Ramsjön (59.837564N, 17.219753E), and from the shore in Hanelundssjön (59.903604N, 17.140360E) to establish *G. semen* cultures. These humic lakes in central Sweden regularly experience summer blooms of this microalgal species (miljodata.slu.se). Single *G. semen* cells were isolated by micromanipulation from the plankton net samples under an inverted Nikon Eclipse Ts2R microscope using 100–200 × magnification, washed three times in sterile medium and transferred to individual wells with a 1:1 mixture of 0.2 μm filtered lake water and Modified WC (MWC) medium [23]. When the clonal cultures started growing, they were moved to pure MWC medium in 40-mL tissue culture flasks (VWR). The cultures were kept in a 12:12-h light to dark cycle at 18 °C.

Upon microscopic observations of small ciliates feeding on *G. semen* cells in natural water samples from the lakes Siggeforasjön and Ramsjön in the following summer, individual ciliate cells were isolated by micromanipulation under an inverted Nikon Eclipse Ts2R microscope using 200–400 × magnification, washed three times in sterile medium, and transferred into *G. semen* cultures in 96-well plates. After clonal ciliate cultures were established, they were kept in 40-mL flasks under the same culturing conditions as described above. The ciliates were fed every one to three weeks with new prey when the *G. semen* culture was depleted from the flask. Dense ciliate cultures were collected on 0.2-μm membrane filters after their *G. semen* prey cultures were nearly depleted and stored at − 80 °C for sequencing.

Aliquots of each ciliate culture were preserved with Lugol’s iodine solution and the length and width of 20 cells each was measured under a Leica DM IL LED microscope with 200 × magnification using a QIclick™ Digital CCD Camera (Bayer Mosaic) and the software Image-Pro Plus 7.0 (Media Cybernetics). The volume of the cells was calculated using the following formula for a prolate ellipsoid:

1 $$V = 4/3\pi *{\mathrm{length}}*{{\mathrm{width}}}^{2}$$

Significant difference in cell volume between the two ciliate strains was tested with a two-sample *t*-test in R base (v. 3.6.3).

### Phylogenetic Analyses of the Ciliate Cultures

To taxonomically identify the ciliate cultures, their DNA was extracted from the filters using the DNeasy PowerSoil Pro kit (Qiagen). The V4-V5 fragment of the 18S rRNA gene was amplified in 20 μL reactions using the DreamTaq Green PCR Master Mix (Thermo Scientific) and the primers 574*f and 1132r [24], which contained attached Illumina adaptors, at a final concentration of 0.25 μm. The PCR was carried out with a 2720 Thermal Cycler (Applied Biosystems) with the following cycling settings: 95 °C for 3 min, followed by 20 cycles of 95 °C for 30 s, 40 °C for 30 s, and 72 °C for 45 s, ending with a final elongation step at 72 °C for 5 min. The amplified products were purified with MagSi-NGS^PREP^ Plus beads (MagnaMedics). The PCR products were reamplified using individual combinations of forward and reverse index primers with Illumina handles to enable multiplexing. The amplicons were paired-end sequenced using an Illumina MiSeq platform to account for potential contamination with remaining prey DNA.

The amplicon sequencing data were processed with the DADA2 pipeline [25] in R-3.6.3 [26]. At most three errors in the forwards reads and six errors in the reverse reads were allowed during filtering, while 21 bp were trimmed from the 5′-end of the forward and 54 bp from the reverse reads to remove primers and low quality bases. The minimum allowed read length was set to 235 bp and the quality threshold to Q2. After dereplicating forward and reverse reads, the DADA2 pipeline identified amplicon sequence variants (ASV) in the dataset. The forward and reverse reads were merged with a minimum overlap of 5 bp and chimeras were identified and removed based on matches with combinations of 3′- and 5′-segments of different sequences. The taxonomy of the ASVs was assigned with the naïve Bayesian classifier method implemented in DADA2 based on the PR_2_ database [27].

Sequences of the ciliate genus *Urotricha* [18] and related Prostomatea were collected from the NCBI database and aligned using Muscle [28] in Unipro UGENE v.41.0 [29] for phylogenetic placement of the newly established ciliate cultures. Positions with less than 10% coverage were trimmed from the alignment. A phylogenetic tree was built with RAxML version 8.2.12 [30] using the GTRGAMMA mutation model and a sequence of *Strombidium purpureum* as outgroup. The tree was visualized using the software FigTree v1.4.4 [31].

### Grazing Experiment

To account for the effect of intraspecific diversity on grazing, three strains of *G. semen* (HL3E12, SH2A6 & SF1A8) were selected as prey for two ciliate strains (E8 & F8). The *G. semen* cultures originated from Hanelundssjön (HL), Stora Hålsjön (SH), and Siggeforasjön (SF), while both ciliate strains were isolated from Siggeforasjön. The cell concentrations of the chosen cultures were calculated from microscopic counts (Leica DM IL LED at 100 × magnification) of aliquots preserved with Lugol’s iodine solution in Utermöhl chambers. The ciliate cultures were filtered through a 25 μm mesh to remove *G. semen* cells of the old prey cultures prior to the microscopic counts. For the experiment, *G. semen* strains were inoculated from exponentially growing cultures into a final volume of 20 mL in 25-cm^2^ tissue culture flasks (VWR) 24 h prior to the start of the experiment (i.e. the addition of the ciliate) to allow for acclimation of the fragile cells to the experimental conditions. Final cell concentrations corresponded to 1000 prey cells mL^−1^ and 200 ciliate cells mL^−1^. Monocultures were set up for each *G. semen* strain by replacing the volume from the ciliate culture with pure MWC medium.

The experimental flasks were kept at 18 °C with a 12/12-h light cycle. Every second day, a 1-mL aliquot was taken from every flask, immediately stained with Lugol’s iodine solution, and the number of *G. semen* and ciliate cells was counted as described above. The experiment was stopped after 20 days when several *G. semen* cultures were depleted.

### Analyses of Grazing Experiment

Linear growth rates (*μ*) were calculated for *G. semen* (in monoculture and with grazers) and the ciliate strain E8 following the formula:

2 $$\mu = \left({e}^{slope}\right)-1$$

When *G. semen* was growing together with ciliate F8, only the time period showing a linear decrease in microalgal cell concentrations was included (see Fig. 3). As ciliate strain F8 followed an exponential growth curve in contrast to strain E8, exponential growth rates (μ) were calculated for this strain following the formula:

3 $$\mu = \frac{\left(ln{N}_{t}-ln{N}_{0}\right)}{t}$$

where *N*_0_ and *N*_*t*_ are the cell concentrations (cells/mL) at day 0 and *t* days later. Differences between mean growth rates between the two ciliate strains were tested using a one-way ANOVA in R base (v. 3.6.3).

Ingestion rates (*I*) for ciliate strain E8 were calculated between all sampling time points (i.e. every two days) from the reduction in prey concentration in the grazing treatment compared to the prey monocultures, as in Heinbokel [32]. For strain F8, ingestion rates were only calculated between sampling time points that showed a linear decrease in *G. semen* cell concentrations and exponential growth of the ciliate.

4 $$I = {B}_{avg}*F$$

*B*_*avg*_ is the average prey cell concentration (cells mL^−1^) in the grazing treatment and *F* is the clearance rate, respectively, calculated as follows:

5 $${B}_{avg} = \frac{\left({B}_{1}-{B}_{0}\right)}{\left(ln{B}_{1}-ln{B}_{0}\right)}$$

6 $$F = \left(k-g\right)/P$$

in which *k* is the growth rate of the prey in the prey monoculture, *g* is growth rate of prey in the grazing treatment, and *P* is the average ciliate concentration in the grazing treatment (calculated as *B*_*avg*_). Differences between the mean growth rate of *G. semen* in the monocultures and the mean ingestion rates of the two ciliate strains were tested with a one-way ANOVA and a Tukey HSD post-hoc test in R base (v. 3.6.3). *p*-values < 0.05 were considered as significant in all statistical analyses.

The impact of grazing on *G. semen* concentrations across all time points compared to the control treatments was tested for both ciliate strains using linear mixed-effect models (lmer) from the R package lme4 [33]. Models with different fixed and random effects, as well as fixed and random slopes and intercepts were compared with ANOVA in R base (v. 3.6.3) to find the model with the best fit. The best fitting model included as fixed effects an interaction term between ciliate strain (E8, F8, control) and time, and a random effects structure that allowed the effect of time to vary between experimental units (individual combinations of *G. semen* strains and ciliate strains). This model also included random intercepts for time and random slopes for experimental units influenced by time. Estimated marginal means (EMMs) and pairwise comparisons between ciliate strains in this model were computed with the R package emmeans [34].

### Sequencing of Environmental Plankton Communities

To investigate the microzooplankton community occurring during *G. semen* blooms, lake water samples were taken with a 1L Ruttner sampler at 0.5 m depth from the same four lakes when the plankton net samples were taken for establishment of *G. semen* cultures (summer 2020). All 45 samples collected on 28 different days from late May to late September were filtered through 100 μm mesh to remove large zooplankton. Volumes of 400 to 600 mL from this sample were filtered through a 10 μm polycarbonate membrane filter with 48-mm diameter (Supor, Pall Laboratory) to collect large protist cells, while the flow-through with the smaller size fraction was collected on a 0.2-μm filter (Whatman, GE Healthcare). The filters were stored at − 80 °C until further processing.

The DNA was extracted from one half of each environmental sample filter using the DNeasy PowerSoil Pro kit (Qiagen) and the V4-V5 fragment of the 18S rRNA gene was paired-end sequenced on an Illumina MiSeq platform as described above. The environmental amplicon sequencing datasets from the lakes Stora Hålsjön, Siggeforasjön, Hanelundsjön, Ramsjön and additionally Erken [35] in central Sweden were searched for reads matching the 18S rRNA gene sequence of the ciliate isolates. Relative read abundance of *G. semen* and the ASV of the ciliate isolates in these samples were plotted against each other with the R package ggplot2 [36].

## Results

### Taxonomic Identification of Ciliate Cultures

**Fig. 1:**
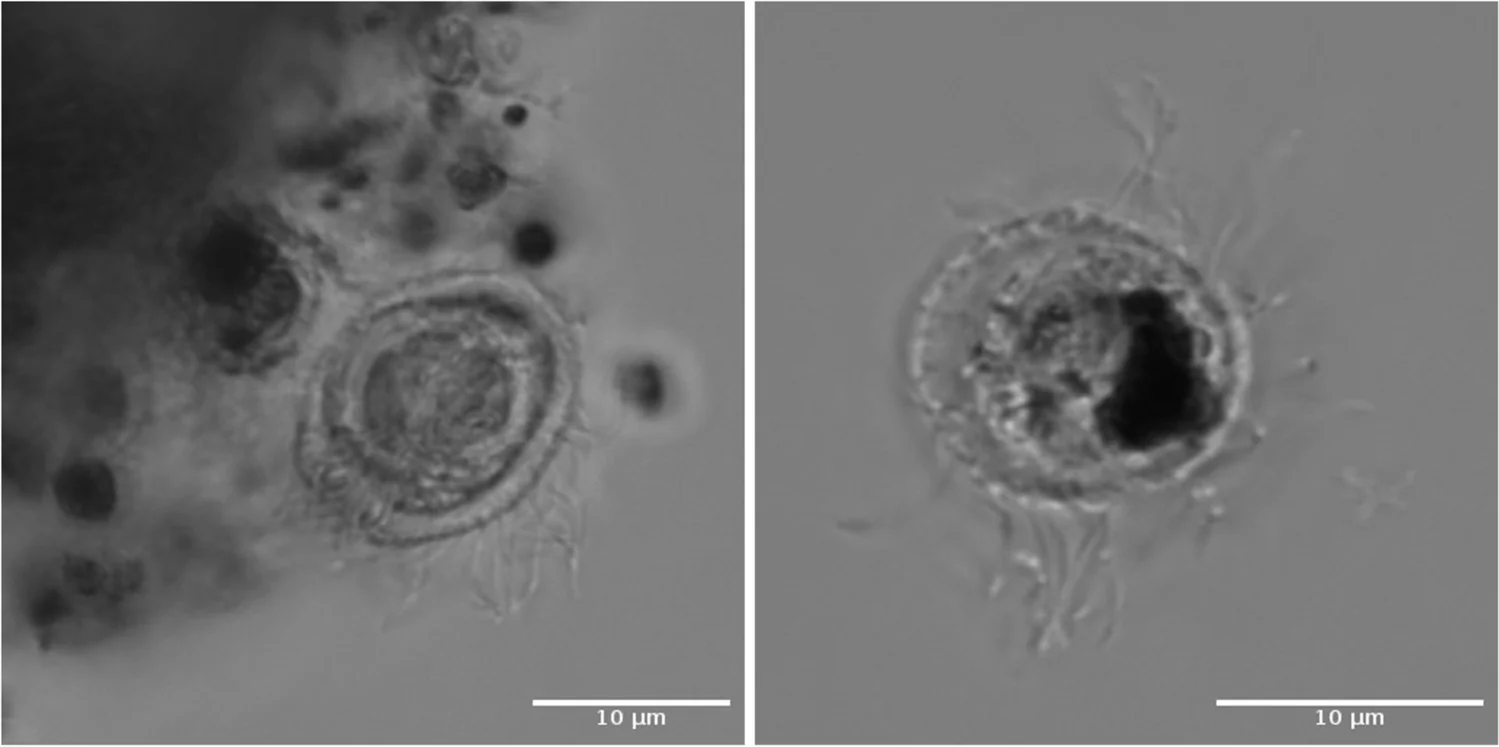
Microphotographs of ciliate isolate E8 using 63x/NA1.2 water magnification with a Zeiss LSM 700 confocal microscope

The final two ciliate cultures (E8 & F8) were both isolated from lake Siggeforasjön. The ciliate cells were ovoid (Fig. 1), and their volume, approximated as a prolate ellipsoid, differed significantly between the two strains used in the experiment (two-sample *t*-test: *t* = 5.13, df = 38, *p*-value = 8.85 × 10^−06^). Strain E8 had a mean cell length of 16.9 μm and a mean width of 11.8 μm, resulting in a mean volume of 10,280 μm^3^. Strain F8 reached on average only 5663 μm^3^ with mean cell length and width of 13 μm and 10 μm, respectively.

**Fig. 2:**
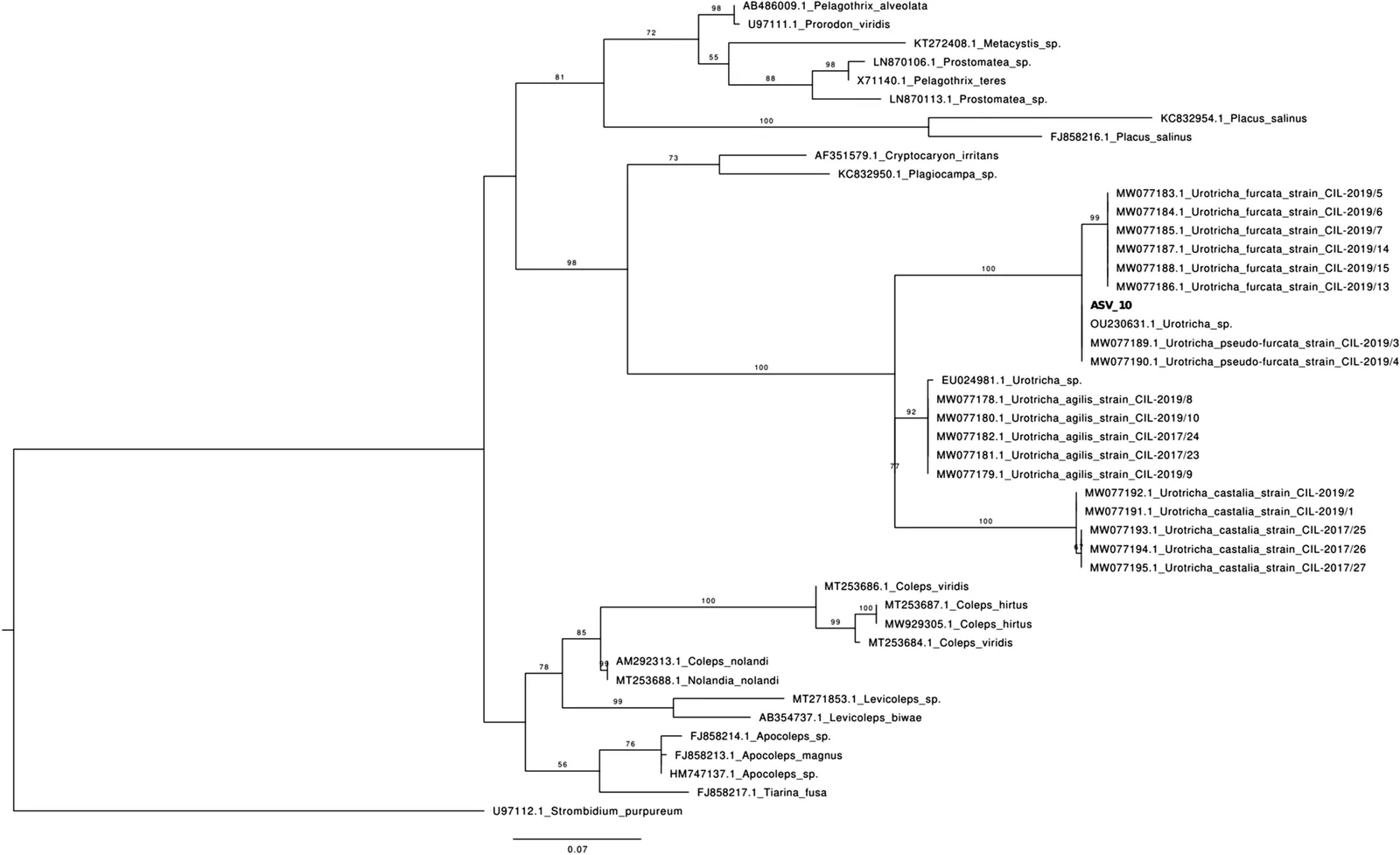
Maximum likelihood tree based on the V4-V5 region of the 18S rRNA gene placing the ciliate isolates (ASV_10) into the species *Urotricha pseudofurcata*. Bootstrap values > 50% indicated on branches

The amplicons of both ciliate cultures were dominated by the same ASV, which was identified as CONTH_4 using the naïve Bayesian classifier method implemented in DADA2 based on the PR_2_ database [27]. Based on blastn search of the 18S rRNA gene fragment against the NCBI database, the ciliate isolates were found to be closely related to *Urotricha sp*. Phylogenetic analyses of the sequences revealed that the ciliate isolates likely belong to the species *Urotricha pseudofurcata* (Fig. 2) based on the work in Frantal et al. (2022).

### Grazing Behavior on *G. semen*

The ciliate isolates showed a very noticeable grazing behavior when targeting *G. semen*, meaning that the ciliates attacked the cell membrane until cytoplasm and organelles spilled out (see video in supplementary material). These smaller particles were then sucked in by the predators. Several ciliate cells usually attacked the same *G. semen* cell simultaneously resulting in rapid disintegration of the entire prey organism.

### Grazing Experiment

**Fig. 3:**
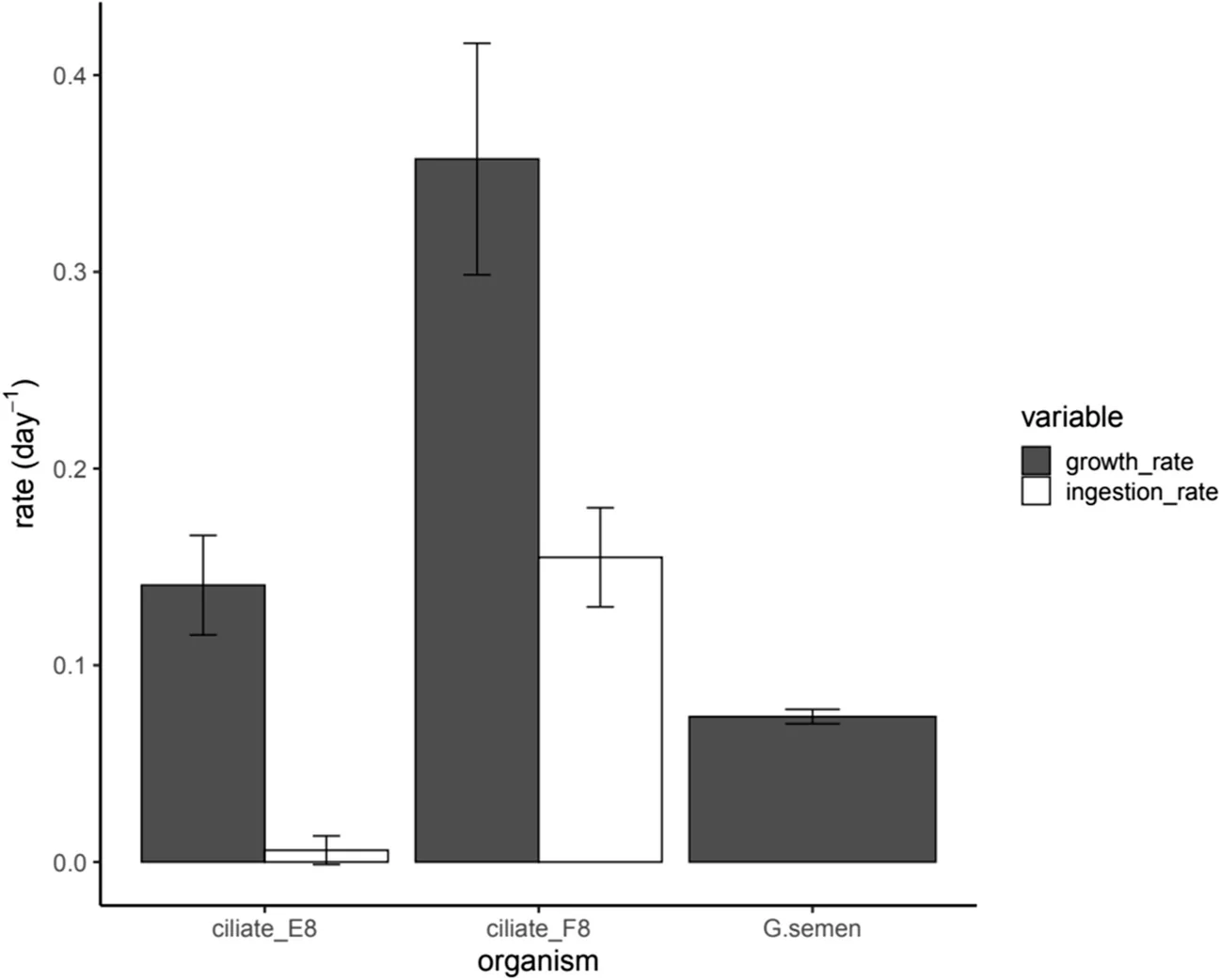
Growth rates of *G. semen* monocultures and the two ciliate strains as well as their ingestion rates in the grazing experiment

The two ciliate strains showed significantly different growth rates in the three *G. semen* cultures (one-way ANOVA: *p* = 0.004). The mean growth rate of E8 was 0.14, while strain F8 displayed a mean growth rate of 0.36 (Fig. 3). The mean growth rate of *G. semen* in the monocultures was 0.074. The mean ingestion rates (E8: 0.006 day^−1^, F8: 0.155 day^−1^) differed significantly between the two ciliate strains (Tukey HSD: *p* < 0.001), and the ingestion rate of ciliate strain F8 was significantly higher than the growth rate of *G. semen* (Tukey HSD: *p* = 0.0015). In contrast, the ingestion rate of ciliate strain E8 was significantly lower than the algal growth rate (Tukey HSD: *p* = 0.0038).

**Fig. 4:**
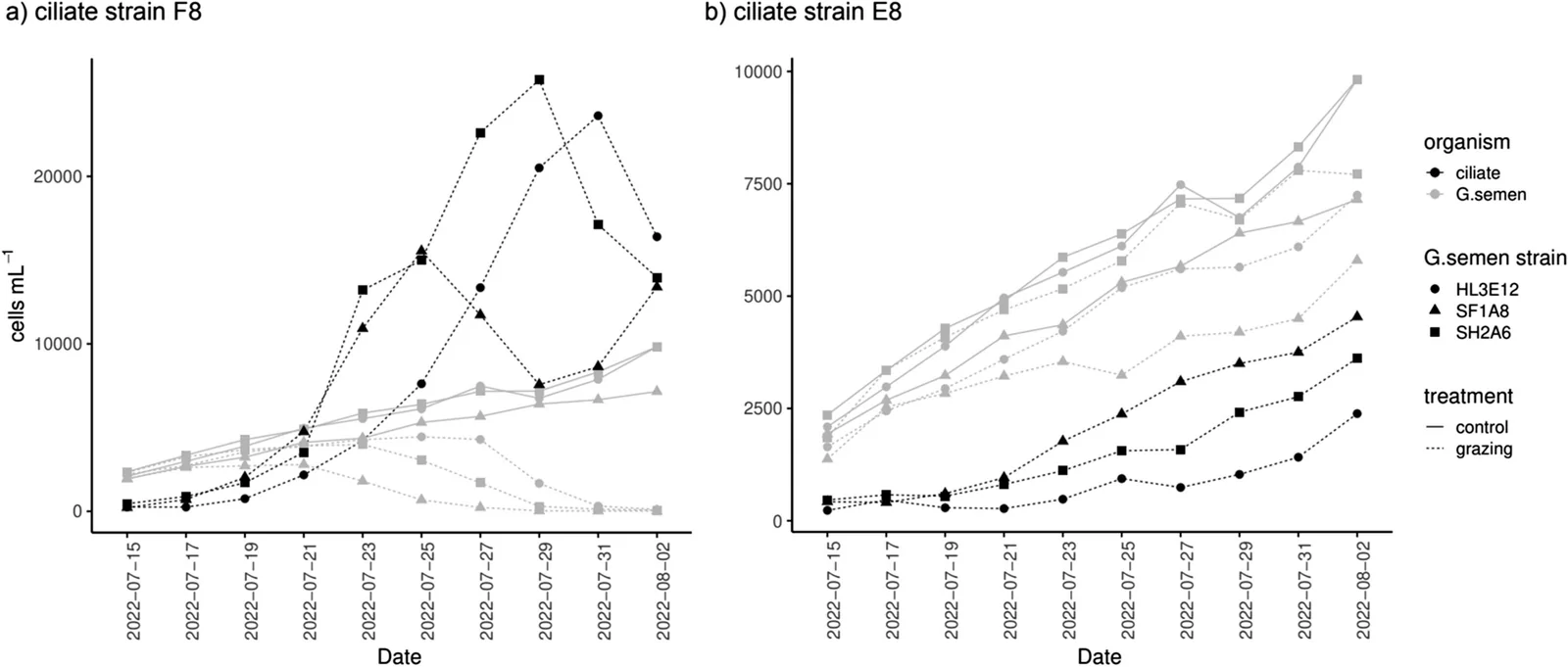
Grazing experiments with the ciliate strains F8 (**a**) and E8 (**b**), and three different *G. semen* strains. Ciliate cell concentrations are indicated in black, while *G. semen* cell concentrations are displayed in gray. *G. semen* cell concentrations in the control treatments without ciliate grazers are represented as solid lines. Please, observe the different scales on the y-axes

The linear mixed-effects model revealed a significant impact (lmer: *p* < 0.05) of the control treatment, grazing by ciliate strain F8, time, and the interaction of grazing by F8 and time on *G. semen* cell concentrations. Over time, *G. semen* abundances differed significantly between the control and the grazing treatment with ciliate strain F8 (EMMs: df = 6, *p* = 0.0045), as well as between the two grazing treatments (EMMs: df = 6, *p* = 0.0235). The strong grazing pressure by ciliate strain F8 resulted in near depletion of all three *G. semen* strains over the course of 20 days (Fig. 4a, HL3E12: 104 cells/mL, SH2A6: 65 cells/mL, SF1A8: 15 cells/mL). This decline in *G. semen* cell concentrations co-occurred with a rapid increase in cell concentrations of the ciliate strain F8 reaching approximately 23,600, 25,800 and 15,500 cells/mL in the three different *G. semen* cultures. Differences in *G. semen* concentrations between the control and the grazing treatment with ciliate strain E8 were less pronounced (Fig. 4b) and not significant (EMMs: df = 6, *p* = 0.326).

### Presence in Environmental Samples

**Fig. 5:**
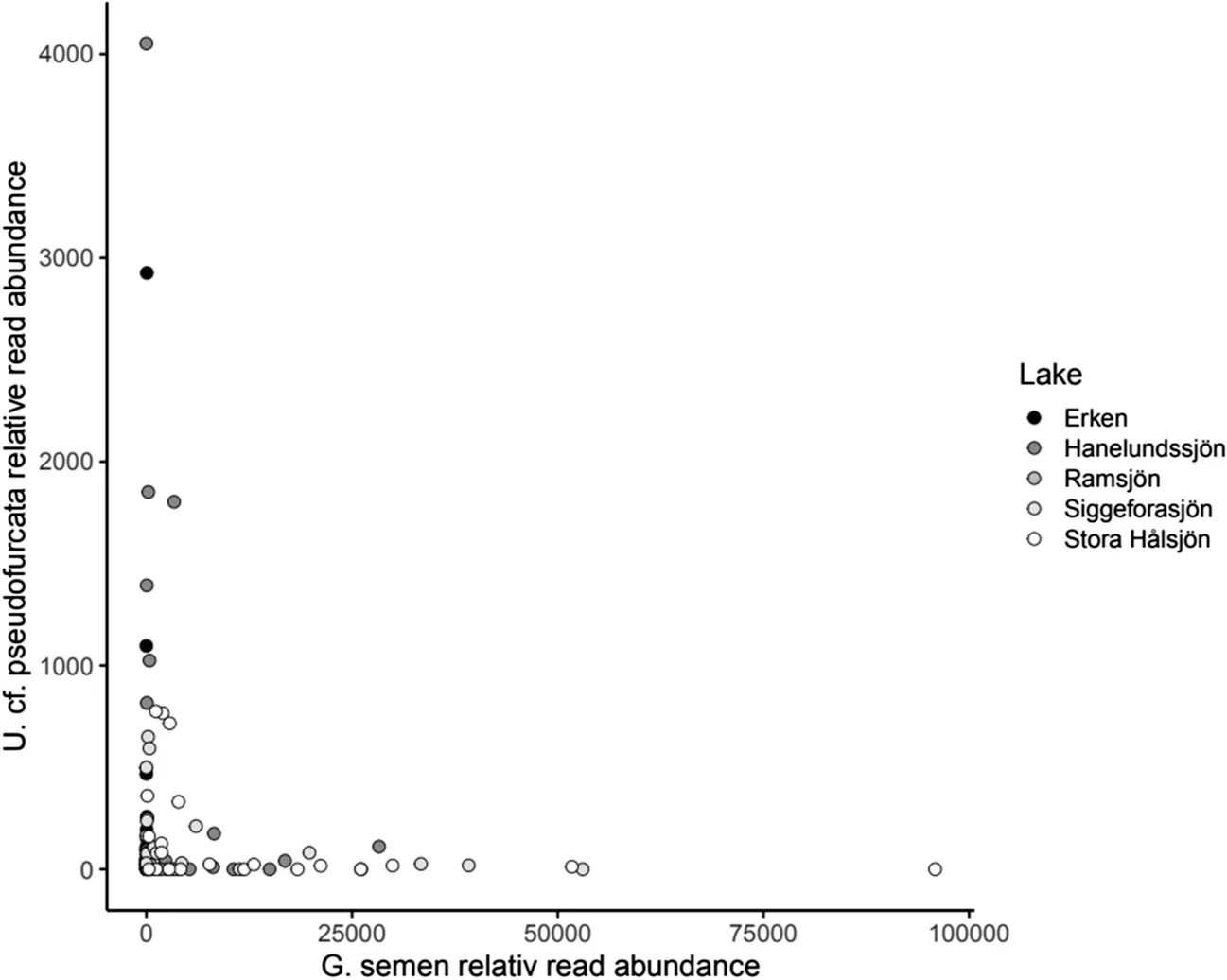
Scatterplot illustrating the co-occurrence of reads from the ciliate *Urotricha* cf. *pseudofurcata* and the microalga *Gonyostomum semen* in multiple samples from five different lakes

Reads of the ASV from the ciliate cultures were found in samples from all five lakes including Erken, where *G. semen* does not occur. The ciliate ASV was mainly present in the small size fraction (0.2–10 μm), while *G. semen* only occurred in the large size fraction (10–100 μm). Plotting the relative read abundance of the two species against each other indicated opposing abundances in the lakes. When *G. semen*’s relative read abundance was high (> 20,000 reads), the relative read abundance of *Urotricha* cf. *pseudofurcata* usually was low (< 5000 reads) and the other way around (Fig. 5).

## Discussion

In this study, we describe a small ciliate species that preys on the large, bloom-forming phytoplankton species *Gonyostomum semen*. Sequencing of clonal cultures of the ciliate tentatively identified it as the species *Urotricha pseudofurcata*. This ciliate species is wide-spread in freshwater and was previously found in 15 different countries [18]. It seems to often occur over wide parts of the water column and can be found throughout the year in some ecosystems [18].

We first noticed the interaction between *G. semen* and the ciliate, when several ciliates were observed by microscopy to attack the algal cells. The repeated attacks caused the microalgal cells to disintegrate in less than one minute (see video in supplementary materials). The attacks of the ciliate cells did not appear to be repelled by release of trichocysts. This could either be due to the small, rapidly moving cells not triggering this defensive mechanism of *G. semen* or that they are able to somehow avoid/ignore the trichocysts. Further research is also need to show if infochemicals [37] attracted the ciliates to the algal cells. Metabolites that are produced by microalgae in response to stressful laboratory conditions [38, 39] could act as such infochemicals and facilitate grazing [40, 41] in culture.

The ingestion rates of 0.006 and 0.15 *G. semen* cells per ciliate and day observed in this study are very low compared to ingestion rates of other *Urotricha* species. Previous studies reported e.g. ingestion rates of 3 to 5 *Cryptomonas* cells per hour in *U. farcta* and *U. furcata* [42]. However, comparisons between these ingestion rates are difficult due to the large differences in size of the prey. In contrast to feeding on small cryptophytes, picocyanobacteria and bacteria, which an individual ciliate cell can completely ingest, multiple ciliates were feeding on each *G. semen* cell individually, ingesting only small parts of the cytoplasm and organelles. Although the ingestion rates were comparatively low, the ingestion rate of ciliate strain F8 was significantly higher than the *G. semen* growth rate resulting in depletion of the prey culture over the course of the experiment.

Despite identical 18S rRNA gene sequences, the differences in cell size and growth rate between the two investigated ciliate strains resulted in noticeably different grazing pressure on the prey populations. The small, fast growing *U.* cf. *pseudofurcata* strain F8 managed to deplete all three *G. semen* strains over the course of the experiment, while *G. semen* cell concentrations were not significantly reduced by grazing from the larger ciliate strain E8 compared to the control treatments. These large phenotypic differences between the two strains likely illustrate the high plasticity of this ciliate species and need to be considered when evaluating its role in aquatic food webs. Cell size can often be considered as a master trait, which influences metabolic rates, growth rates, resource acquisition, and susceptibility to grazing [43]. The small cell size of ciliate strain F8 might thus facilitate its high growth rate. Conclusions for the overall effect of grazing by *U.* cf. *pseudofurcata* on *G. semen* have to be drawn, however, with caution, as the predator and prey concentrations at the end of the experiment exceeded cell concentrations observed in nature [7, 18].

Predator–prey-interactions between *U.* cf. *pseudofurcata* and *G. semen* likely also take place in nature, as the environmental sequencing data confirm their co-occurrence in several humic lakes. Noticeably, the two species often displayed opposing relative abundances. This observation might suggest that *G. semen* can only thrive and reach high cell concentrations during low abundances of *U.* cf. *pseudofurcata*. However, amplicon sequencing data of environmental communities do not represent quantitative data and potential correlations between species relative abundances need to be considered with caution. Additionally, *U.* cf. *pseudofurcata* and *G. semen* cells were not present in the same size fraction, and were thus sequenced in separate samples. Their relative read abundance was therefore not directly proportional to each other. Nevertheless, general trends such as presence and absence, as well as high and low abundance, are usually representative for the overall community composition [44, 45]. In the future, predator–prey dynamics between *U.* cf. *pseudofurcata* and *G. semen* could potentially be revealed in nature by more frequent (e.g., daily) sampling and more quantitative methods.

The interaction between *U.* cf. *pseudofurcata* and *G. semen* in nature is likely impacted by multiple different factors such as plankton community composition and environmental conditions. For instance, mesozooplankton might prey on the ciliates, depending on their cell size, and reduce the grazing pressure on *G. semen* [46]. Furthermore, the co-occurrence of other potential prey species could influence the interactions between *U.* cf. *pseudofurcata* and *G. semen* [47, 48]. Previous studies have suggested a wide prey range in prostomatid ciliates [16, 19] and grazing on small sized prey species, which can be directly ingested, might be more efficient than attacking large *G. semen* cells. For instance, *U.* cf. *pseudofurcata* in lake Erken likely grazes on small *Cryptomonas* cells or bacteria instead of *G. semen*. Regrowth of the ciliates after the depletion of *G. semen* in our experiments could also be due to ingestion of bacteria benefiting from released organic matter [49–51], or even cannibalism [52]. However, the growth of preferred phytoplankton prey species is likely reduced during *G. semen* summer blooms [53–55], while the encounter rate with the freshwater raphidophyte will be very high. In such a scenario, ciliate grazing on *G. semen* might be very common. The nightly vertical migration of *G. semen* below the thermocline into the often anoxic hypolimnion [56–58] might, however, reduce this predator–prey interaction. Although ciliates also perform diel vertical migration [59, 60], Peltomaa et al. [61] showed that algivorous ciliates, including *Urotricha*, disappear from the hypolimnion of a boreal, humic lake when it became anoxic in summer. At the same time, peak concentrations of *G. semen* (> 50 mg chl *a* m^−3^) were observed in the hypolimnion. Grazing might thus mainly occur during the day when *G. semen* is present in oxic surface waters.

In any case, our results show that *Urotricha* cf. *pseudofurcata* may play an important role in aquatic ecosystems that are regularly dominated by *G. semen*. The freshwater raphidophyte can form extensive, nuisance summer blooms [2, 7], which could get reduced by efficient grazing of this small ciliate species allowing the development of a more diverse phytoplankton community. Furthermore, the nutrients and organic carbon that are released when the ciliates attack *G. semen* cells, are available for remineralization by bacteria [62]. In the absence of grazers, most organic carbon produced by this phytoplankton species would be buried in the sediment and would not be available for higher trophic levels in the water column [54]. However, the remineralized dissolved nutrients will be transported back to the food web via the microbial loop [62]. *Urotricha* cf. *pseudofurcata* represents therefore a potential key link between primary producers and larger consumers in humic freshwater ecosystems with *G. semen* summer blooms.

Our study shows that these small prostomatid ciliates have a much wider prey range than previously described and that they also need to be considered as grazers of microplankton. Future studies need to investigate if these ciliates can also feed in a similar manner on other large phytoplankton species, which do not possess a sturdy cell wall, such as naked dinoflagellates and Euglenophyceae. For instance, a previous study provided evidence for increased mortality of the rotifer *Keratella quadrata* in the presence of *Urotricha furcata* and *U. farcta* [63]. The authors suggested that the adverse effect of the ciliates on *K. quadrata* was likely mediated by chemical defenses of *Urotricha* against rotifer predation, but reverse predator–prey interactions, similar as observed in this study, have to be considered now as well. Overall, this study highlights that grazers do not necessarily have to be larger than their prey organisms and predator–prey interactions might be found among surprising size classes in plankton communities.

## Supplementary Information

Below is the link to the electronic supplementary material.

Supplementary file1 (MP4 5857 KB)

## Data Availability

The sequenced 18S rRNA gene fragment of the ciliate isolates can be accessed in GenBank under the accession number OP684302. The raw amplicon sequence reads of the environmental samples are available in the Sequence Read Archive (SRA) of NCBI in BioProject PRJNA1004546 under the accession numbers SAMN36943666 to SAMN36943755. The dataset with the cell counts from the grazing experiment is archived in DiVA and can be found under the URN urn:nbn:se:uu:diva-511093 as well as the DiVA id diva2:1,795,087.
